# A Project-based Curriculum for Driving Organization-wide Continuous Improvement

**DOI:** 10.1097/pq9.0000000000000138

**Published:** 2019-02-12

**Authors:** Lory D. Harte, Mamta Reddy, Lisa K. Marshall, Kevin J. Mroczka, Keith J. Mann

**Affiliations:** From the Departments of * Improvement Academy; † Performance Improvement; ‡ Strategic Planning; § Pediatrics, Children’s Mercy Hospital, Kansas City, Mo.; ¶ University of Missouri- Kansas City School of Pharmacy, Kansas City, Mo; ‖ University of Missouri - Kansas City School of Medicine, Kansas City, Mo

## Abstract

**Background:**

Creating the capacity and capability for meaningful improvement in healthcare quality is a challenge many organizations face. Before 2012, Children’s Mercy sponsored 20 leaders to obtain advanced improvement training from peer organizations. Recognizing an opportunity to build upon this momentum, we developed an organization-wide curriculum for teaching continuous improvement.

**Methods:**

A steering committee was created in 2011 to define, advise, and oversee education in improvement science. We agreed upon a framework for improvement, a program name [Continuous Quality and Practice Improvement (CQPI)], and a phased curriculum development approach, beginning with a project/experiential learning based course (Team CQPI). Course evaluation for Team CQPI consisted of a standard evaluation of objectives, pre- and post-course assessment, qualitative feedback, and serial assessment of project progress using the Team Assessment Score (TAS). The curriculum committee monitored improvement.

**Results:**

From 2012 to 2017, 297 people participated in the project-based course, completing a total of 83 projects. TAS improved throughout the 4-month project-based course, from an average starting score of 1 (“forming a team”) to 2.7 (“changes tested”). The average TAS at 12 months following completion of the Team CQPI course was 3.5 (“improvement”) out of 5.

**Conclusions:**

Development of a comprehensive curriculum for driving continuous improvement has resulted in a measurable change in TAS scores representative of local improvement efforts.

## INTRODUCTION

In 2001, the Institute of Medicine identified a chasm in the quality of health care in the United States and called for a fundamental change.^1^ Subsequent literature suggested that patients may only receive optimal care 50% of the time.^2,3^ Also, the quality of care may vary just because of the location in which a patient chooses to live.^4^ Less than the optimal performance across multiple acute care and preventive care measures and multiple geographic locations suggest the need for broad-based quality improvement (QI) efforts led by health care professionals. Despite this need, many health care providers have not been exposed to QI concepts or may not have the knowledge or skills to succeed in their improvement efforts.^5^ Also, health care organizations may struggle to provide the necessary resources to support the widespread QI efforts.^6^

Improvement capacity is related to creating the structure, processes, and QI specialists to support improvement teams. Improvement capability, on the other hand, is related to developing leaders to effectively conduct improvement projects that result in a sustainable, measurable change.^7^ Perla et al^8^ identified 4 primary factors that drive large-scale implementation of change: planning and infrastructure; individual, group, organizational, and system factors; the process of change; and performance measures and evaluation. Also, large-scale improvement may not occur until the front-line staff and middle management incorporate problem-solving and change management behaviors at the bedside as an integral part of providing patient care.^9^

In 2011, leaders identified an opportunity to capitalize on their improvement training obtained at a peer organization. As a result, resources were allocated to develop an organization-wide curriculum for teaching continuous improvement. This study describes the results of implementing an organization-wide, project-based continuous improvement education course in a free-standing children’s hospital.

## METHODS

Children’s Mercy Kansas City is a comprehensive pediatric academic health center that employs over 8,000 pediatric specialists, physicians-in-training, nurses, allied health professionals, and ancillary staff. Physicians and staff care for pediatric patients in 2 hospital campuses, a 315-bed, full-service facility in Kansas City, Mo. and a smaller 53-bed facility in Overland Park, Kans. The primary catchment area includes several additional urgent care and ambulatory sites.

Between 2008 and 2011, the hospital sent 22 physician, nursing, pharmacy, and quality leaders to the Advanced Training Program at Intermountain Healthcare, at the cost of approximately $135,000, with the goal of increasing the organization’s improvement capacity and capability. In 2011, quality leaders and executives committed to building and sustaining an internal training program for QI. They created a steering committee consisting of executives, physician leaders, nursing leaders, and educational leaders. Five of the 17 steering committee members previously attended Advanced Training Program, and 8 were trained internally. The committee hired a program director with expertise in QI within health care. Also, they identified administrative support and a physician champion. Finally, they named the program Continuous Quality and Practice Improvement (CQPI). Later, a program manager was hired to support the day-to-day coordination of the program. The annual budget for the program was $250,000, which included operating costs and salaries for 2 employees.

The CQPI education program’s mission was to educate faculty and staff on improvement methods through the direct application of tools that lead to meaningful change while continuously improving the program to meet demand. The goal was to train enough staff to reach a tipping point of proliferation where CQPI-trained staff would start teaching and coaching others, expanding our organization-wide capability for improvement work. The steering committee and CQPI leads began by training mid-level leaders through an intensive, project-based course (Team CQPI).

The CQPI Roadmap, adapted from the Associates in Process Improvement, was the framework used to drive continuous improvement and walk the team through an improvement cycle.^10^ The Team CQPI course included modules related to improvement science, including leading change, problem investigation, designing high-reliability interventions, Plan-Do-Study-Act cycles, sustaining change, and patient safety. QI experts coached the team in the application of project tools during class and in between sessions. Graduates demonstrating an understanding of the methodology became coaches and committed to supporting other coaches through a coaching network.

Participants were selected through application or invitation by senior leadership. Applications were open to teams who identified an improvement opportunity related to their area of practice. Project applications were reviewed and scored based on the following criteria: feasibility; alignment with the Institute of Medicine “Aims for Improvement”^1^; inclusion of adequate measures; inclusion of a specific, measurable, actionable, relevant, and time-bound aim; diversity of the team; completeness of the problem statement; and potential to impact other areas within the organization. Approximately 7 teams consisting of 3–5 members were selected through application each semester. Participants were selected from multiple disciplines. Senior leaders identified up to 10 additional participants who would benefit from the course based on their professional development plan and role within the organization. Approximately 30 participants enrolled in the course, during each of 2 semesters per year. Participants were relieved of their regularly scheduled work for the 56 contact hours of the project-based course.

The program director used both qualitative and quantitative methods to evaluate the curriculum. After each 4-month course, the evaluation scores and participant feedback were used in Plan-Do-Study-Act cycles to improve the curriculum. The Steering Committee determined the overall success of the program through the Team Assessment Score (TAS), which was adapted with permission from the Institute for Healthcare Improvement.^11^ The TAS is a Likert Scale–based assessment of improvement progress for a given project where a score of 0.5 equates to “intent to participate,” a score of 3.0 equates to “modest improvement,” and a score of 5.0 equates to “outstanding, sustained improvement” (Table 1). Data collection occurred at 7 time points: after each of 4 sessions and at 6, 12, and 18 months after completion of the course. Both the team leader and the team’s coach reported a TAS. Also, a survey assessing skill acquisition, skill utilization, and generation of scholarly work was used to assess behavior changes over time. The questions corresponded with a Likert Scale of strongly agree, agree, neutral, disagree, and strongly disagree. Participants received a survey after each session, with 2 reminders 2 weeks apart. An administrative fellow conducted follow-up phone interviews with the leaders of projects who met their aim and/or achieved a TAS of ≥3.

**Table 1.**
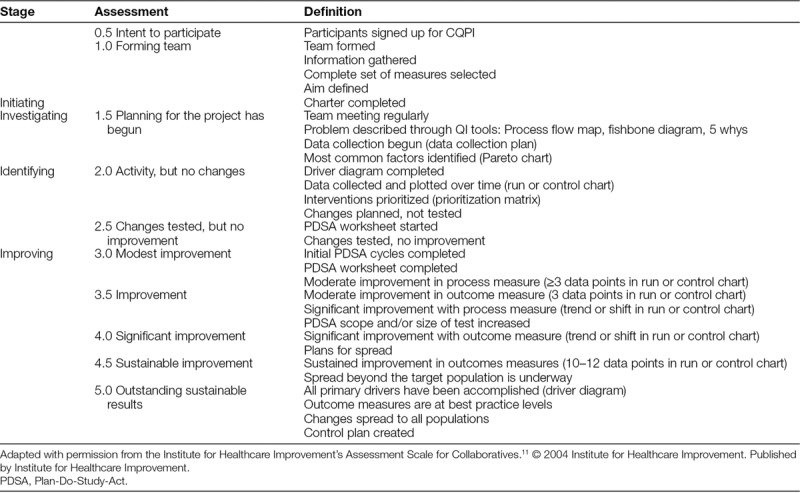
Continuous Quality and Practice Improvement Team Assessment Score

The program director and program manager analyzed TAS through basic statistical calculations of an average, minimum, and maximum for each time point. Also, we aggregated the number and percent of participant responses to each question that utilized a Likert Scale. The program director presented the aggregated data to the Steering Committee quarterly for each semester that the course was offered and across the course as a whole.

The Children’s Mercy Office of Research Integrity reviewed this study and designated it as not human subjects’ research.

## RESULTS

**Fig. 1.**
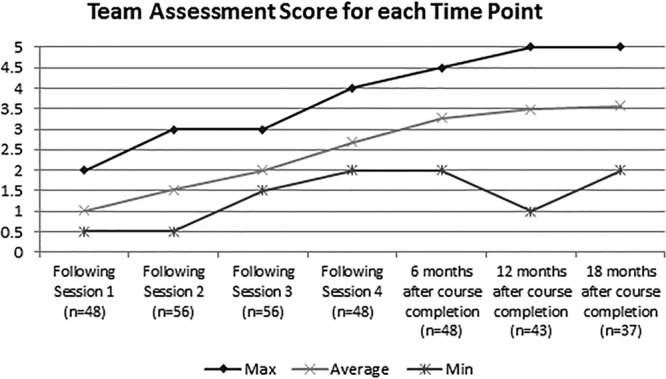
TAS reported by teams who completed the course between Fall 2012 and Spring 2017. The maximum included in this figure is the largest participant reported TAS at each time point. The average is the central tendency of all participant reported TAS at each time. The minimum is the smallest participant reported TAS at each time point.

Eighty-three projects were completed by 297 Team CQPI participants between 2011 and 2017 (Table 2). Project teams reported progress in advancing through the project cycle with an increasing TAS at each time point. TAS scores improved throughout the 4-month project-based course, from an average starting score of 1 (forming a team) to 2.7 (changes tested). The average TAS at 12 months after completion of the course was 3.5, representing improvement (Fig. 1). Seventy-six percent of respondents surveyed 12 months following course completion reported a TAS of ≥3. The average survey response rate was approximately 63%.

**Table 2.**
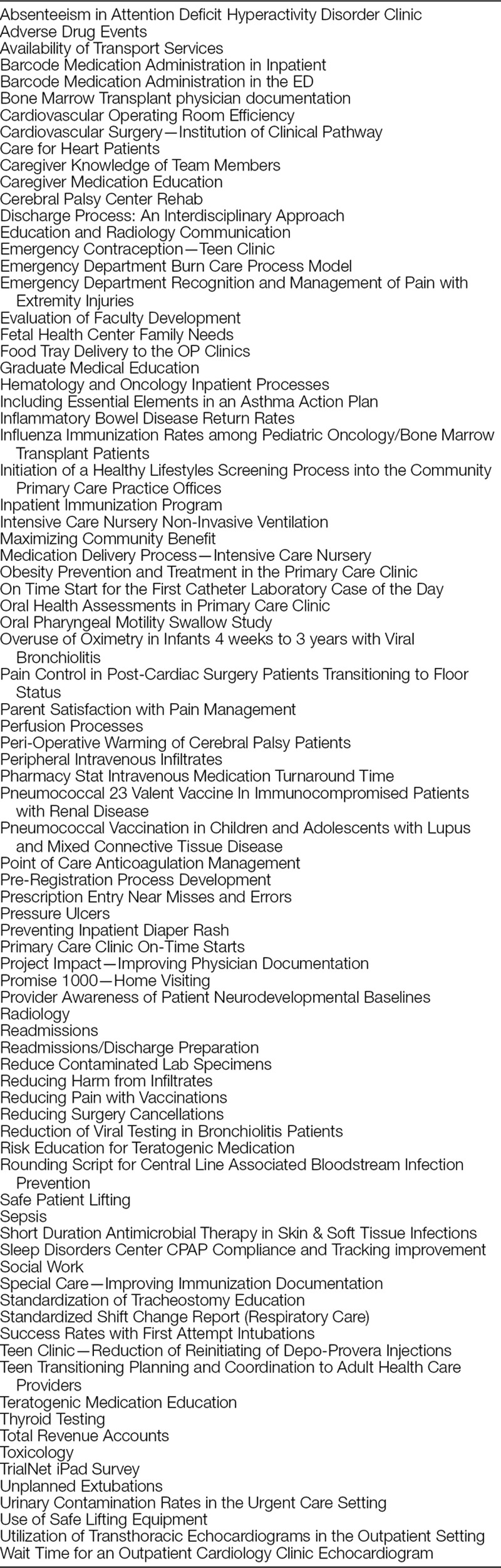
List of Project Names

Overall, participants agreed that the course met the learning objectives (Table 3). Similarly, the evaluation of most presenters was positive. Participants appeared to like the schedule and timing of sessions. They also felt the content of the course was informative and interesting for all semesters. When asked at 6, 12, and 18 months after completion of the course whether they had completed their improvement project, on average, more than half of the participants responded that they had done so or would do so in the next 3 months. Also, most of the participants applied the skills learned in Team CQPI on another improvement project and would recommend the course to a colleague interested in learning about the tools and methodology of QI. Although only a small number of participants have published their project,^12–16^ many report that they intend to do so or have presented an abstract or poster of their work at a regional or national conference (Table 4).

**Table 3.**
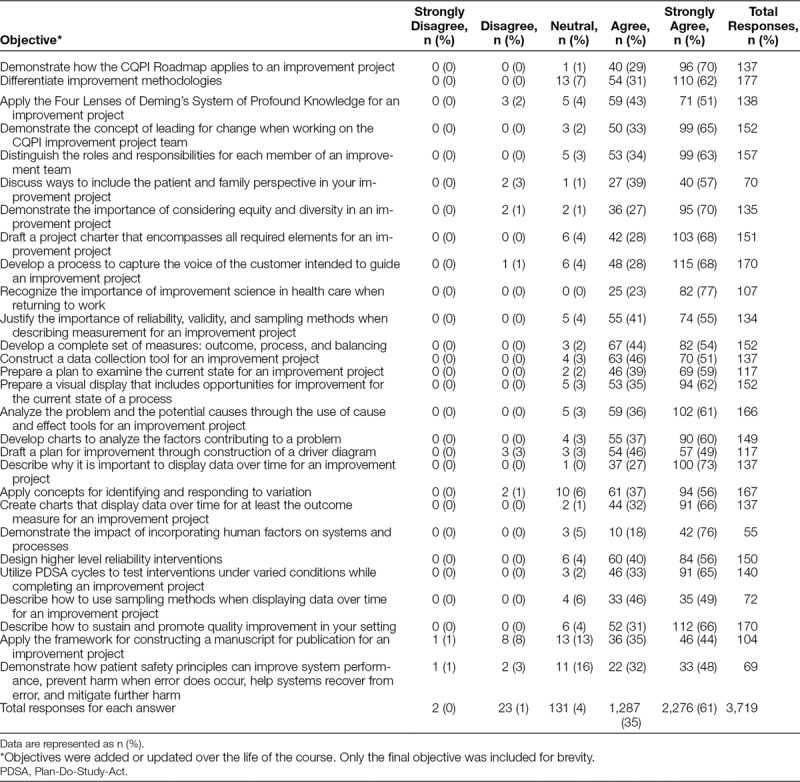
Course Evaluation of the Degree to Which Objectives Were Met

**Table 4.**
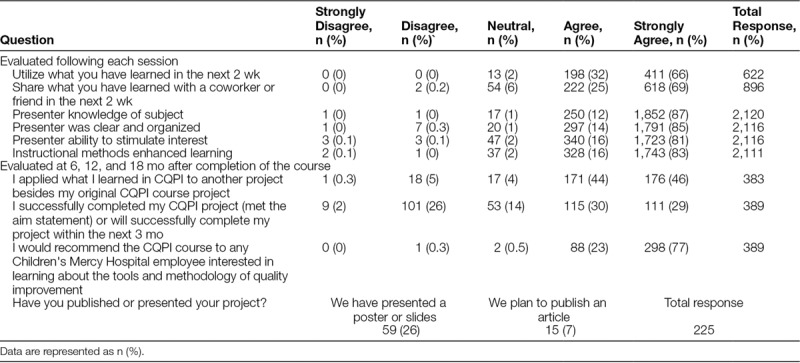
Course Evaluation Results

A majority of participants were physicians, nurses, and allied health professionals (Table 5). Twenty-two participants, who demonstrated an exceptional understanding of the methodology, became coaches. As a result, we developed a coaching network to assist the primary QI coach in effectively supporting future teams. Also, 2 participants were selected to participate in the initial cohort of the Quality and Safety Improvement Scholars Program (Academic Pediatric Association) and 3 went on to obtain further training in advanced QI methods to improve their academic careers. Five participants now lead QI for their respective divisions.

**Table 5.**
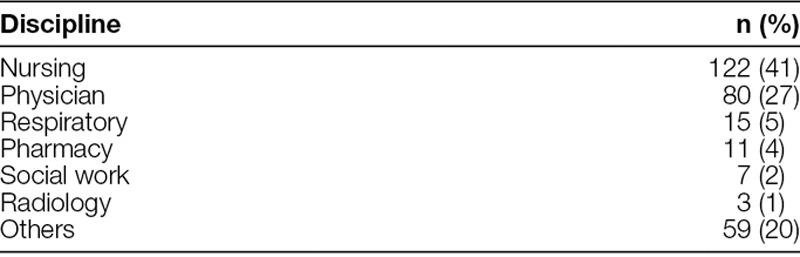
Breakdown of Participants by Discipline

Patient care processes and clinical outcomes have improved as a result of successful projects. These improvements include, but are not limited to, improved assessment of risk for readmission,^12^ reduction in pain related to vaccination,^13,14^ improved oral health,^15^ and shorter wait time for an echocardiogram.^16^ When specifically asked about key factors for successful completion of their projects, project team leaders identified leader support, adequate “protected time” to work on the project, and access to necessary resources such as data, improvement methods, and coaching.

## DISCUSSION

Similar to experience documented in the literature,^8,17^ participants in the project-based CQPI course reported learning, retention, and application of core QI concepts. Participants also reported a change in behavior in the application of skills in their daily work, which is more important than knowledge acquisition alone.^18^ Through assignment of a TAS at each of 7 time points, participants reported an ability to move a project forward both during the course and the months following the course completion with guidance from an improvement coach. Also, and similar to the previous study, a strength of our curriculum design was that the participant could directly apply the constructive coaching feedback to the project.^17^

Published systematic reviews of QI and patient safety programs have found that the most common research design for QI education is a simple pre–post comparison.^19^ The CQPI Steering Committee primarily assessed the course through the success of the projects. Also, the TAS was collected in a time series, making our course unique. The sequential assessment of TAS allowed real-time assessment of the course (after each session) and longitudinal assessment of the overall course. Also, requiring teams to assess their progress helped to reinforce the overall goal of not just knowledge acquisition but also real-time application.

Most previously published improvement curricula included only evaluations of educational outcomes. A systematic review found that curriculum that evaluated both education and clinical outcomes lacked evidence of the relationship between the 2 outcomes.^20^ The nature of the TAS allowed for program evaluation based on the improvement within the system. The project-based CQPI course encouraged an interdisciplinary approach to QI that resulted in not only meaningful learning and improvement for those involved but also in improved processes and patient care outcomes. Leadership support, organizational alignment, and access to resources (including data and QI experts) were strengths of the design that helped drive these local improvements.^7,20^

At an individual level, participants have contributed to building both capacity and capability through coaching within the program and/or within their division. Although the generation of scholarly work and the development of improvement leaders and scholars were not predicted outcomes, similar to other published studies, this is a desirable result of the course and aligns with advancing our academic profile.^17,20^ Also, access to educational programs and courses designed to help junior faculty translate their passion for improvement science into leadership opportunities and academic capital helps to reinforce academic excellence and may help recruit junior faculty in the future.^21^

We noted several limitations in our evaluation design for Team CQPI. One limitation was the use of self-assessment data to measure course effectiveness and acquisition of skills and behavior changes. Self-assessment and monitoring may not always correlate with actual performance; however, the use of internal and external data strengthens the assessment.^22–24^ We evaluated the discrepancies between participant- and coach-reported TAS as a means to facilitate discussion and future learning, but we did not conduct statistical correlations. Also, measuring TAS at all proposed time points did not begin until 2013. A low response rate is also a limitation to the interpretation of results, particularly for those assessments conducted at 6, 12, and 18 months after completion of the course. We would have preferred to measure the impact of the course on clinical outcomes and project-specific improvements, but these are difficult to capture and articulate in aggregate.^17,25,26^ Also, improvements in patient care processes and clinical outcomes usually were not realized until 6–18 months after completion of the course. However, the course-trained influential improvement leaders throughout the organization played a key role in the improvement of strategic priorities, such as a reduction in patient harm from hospital-acquired conditions.

Future work includes incorporating lean thinking into the framework for improvement to take full advantage of the organization’s daily management system. We plan a more rigorous approach to assess learning outcomes and behavior change in future revisions of the course. Finally, the inclusion of supporting courses that dive deeper into focused topics such as patient safety, high reliability, human factors, and improvement research will augment the overall improvement education program.

## CONCLUDING SUMMARY

The development of a comprehensive project-based improvement course resulted in the measurable improvement in selected projects during the course, ongoing improvement after completion of the course, and participant engagement in further improvement efforts after course participation. The Team CQPI course has become the foundation for building improvement capacity and capability within our organization.

## DISCLOSURE

The authors have no financial interest to declare in relation to the content of this article.
